# Cross-Species Permissivity: Structure of a Goat Adeno-Associated Virus and Its Complex with the Human Receptor AAVR

**DOI:** 10.1128/jvi.01484-22

**Published:** 2022-12-01

**Authors:** Edward E. Large, Mark A. Silveria, Onellah Weerakoon, Tommi A. White, Michael S. Chapman

**Affiliations:** a Department of Biochemistry, University of Missouri, Columbia, Missouri, USA; Cornell University

**Keywords:** AAV5, AAVGo.1, AAVR, cell entry, virus receptor, gene therapy, AAV, adeno-associated virus, cryo-EM, electron microscopy, goat, receptor, structure

## Abstract

Adeno-associated virus (AAV) is a small ssDNA satellite virus of high interest (in recombinant form) as a safe and effective gene therapy vector. AAV’s human cell entry receptor (AAVR) contains polycystic kidney disease (PKD) domains bound by AAV. Seeking understanding of the spectrum of interactions, goat AAVGo.1 is investigated, because its host is the species most distant from human with reciprocal cross-species cell susceptibility. The structure of AAVGo.1, solved by cryo-EM to 2.9 Å resolution, is most similar to AAV5. Through ELISA (enzyme-linked immunosorbent assay) studies, it is shown that AAVGo.1 binds to human AAVR more strongly than do AAV2 or AAV5, and that it joins AAV5 in a class that binds exclusively to PKD domain 1 (PKD1), in contrast to other AAVs that interact primarily with PKD2. The AAVGo.1 cryo-EM structure of a complex with a PKD12 fragment of AAVR at 2.4 Å resolution shows PKD1 bound with minimal change in virus structure. There are only minor conformational adaptations in AAVR, but there is a near-rigid rotation of PKD1 with maximal displacement of the receptor domain by ~1 Å compared to PKD1 bound to AAV5. AAVGo.1 joins AAV5 as the second member of an emerging class of AAVs whose mode of receptor-binding is completely different from other AAVs, typified by AAV2.

**IMPORTANCE** Adeno-associated virus (AAV) is a small ssDNA satellite parvovirus. As a recombinant vector with a protein shell encapsidating a transgene, recombinant AAV (rAAV) is a leading delivery vehicle for gene therapy, with two FDA-approved treatments and 150 clinical trials for 30 diseases. The human entry receptor AAVR has five PKD domains. To date, all serotypes, except AAV5, have interacted primarily with the second PKD domain, PKD2. Goat is the AAV host most distant from human with cross-species cell infectivity. AAVGo.1 is similar in structure to AAV5, the two forming a class with a distinct mode of receptor-binding. Within the two classes, binding interactions are mostly conserved, giving an indication of the latitude available in modulating delivery vectors.

## INTRODUCTION

Adeno-associated viruses (AAVs) are small single-stranded DNA viruses that can be repurposed into effective and safe gene therapy delivery vehicles (1). Primate AAV serotypes are the dominant choice for gene therapy. Several structures have been determined, starting with that of AAV2 by X-ray crystallography (2). AAV coding regions consist of two major open reading frames (ORFs), *rep* and *cap*, encoding functions needed in viral replication/DNA packaging and the capsid protein, respectively (3). The Cap ORF encodes phenotypes relevant in tissue tropism and immune recognition (4, 5).

AAV2 was instrumental in the discovery and characterization of a human proteinaceous AAV receptor (AAVR) (6). AAVR is a glycoprotein (N-linked/O-linked) and contains three major protein regions. The N terminus contains a motif at N-terminus with eight cysteines (MANEC), while the central extracellular portion contains five polycystic kidney disease (PKD) domains numbered 1 to 5 from N terminus to C terminus (7). The C terminus has a predicted transmembrane protein domain and cytosolic domain responsible for trafficking AAV through the trans-Golgi network (TGN) (6). Experiments in mouse models and human cell lines indicate the physical interactions between primate AAVs and AAVR receptors of different species can be conserved (6).

For human AAVR, a few structural complexes with AAV are known (8 to 14). The structures consist of AAV1, AAV2, AAV5, or AAV9 serotypes complexed with human PKD variants containing domains 1 and 2 (PKD12) or domains 1 to 5 (PKD1-5). Both structure and mutant data (15, 16) consistently indicate AAV5 is unlike all other AAVs in that its interactions with AAVR are mediated primarily through PKD1 instead of PKD2. Domain-swap mutants show AAV5 interacting exclusively with PKD1 (15), and the cryo-EM structures of complexes with AAVR fragments show that the PKD1 binding site on AAV5 (9, 11) is different from the common PKD2 site for AAV2 and AAV1 (8, 9, 11, 12).

Domain-swap mutants indicate a secondary role for PKD1 with AAV2 and other serotypes (15), but multiple cryo-EM structures reveal only the tightly bound PKD2 at high resolution (8, 12 to 14). It seemed possible that AAV2-like viruses bound not just to PKD2, but also to PKD1, though so weakly that PKD1 had not been seen in the AAV2 structures. It was not as simple as overlaying PKD1 in an AAV5-like position and connecting it, as a single subunit chain, to PKD2 as found in AAV2, because connecting the domains required implausible stereochemistry (11).

AAV gene therapy utilizes primate serotypes of which AAV5 is currently the only known representative with primarily PKD1 interactions. AAV5 was initially isolated from a human penile lesion (17) and is the sole primate AAV5 clade representative. The AAV5 clade also includes AAVGo.1 (18, 19), which was isolated from deceased neonatal domesticated goat ileum (20). Both recombinant AAV5 and AAVGo.1 can transduce at equivalent levels in primate and ruminant cell lines (18, 19). Efficient transduction of primate and ruminant cell lines by both AAV5 and AAVGo.1 represents the broadest divergence between AAV host species that are reciprocally permissive to cross-species infection. Given the strong evidence for AAV5 interactions with PKD1, we investigated potential interactions of AAVGo.1 with AAVR.

Here, binding of AAVGo.1, AAV5, and AAV2 to domain combinations of AAVR is compared by ELISA (enzyme-linked immunosorbent assay). Then, high-resolution structures are determined by cryo-EM of AAVGo.1 alone, and in complex with a PKD12 fragment of AAVR. These are compared to corresponding structures of AAV5 to understand the diversity of interactions that are compatible with productive receptor-binding.

## RESULTS

### AAVGo.1 binds AAVR.

**FIG 1:**
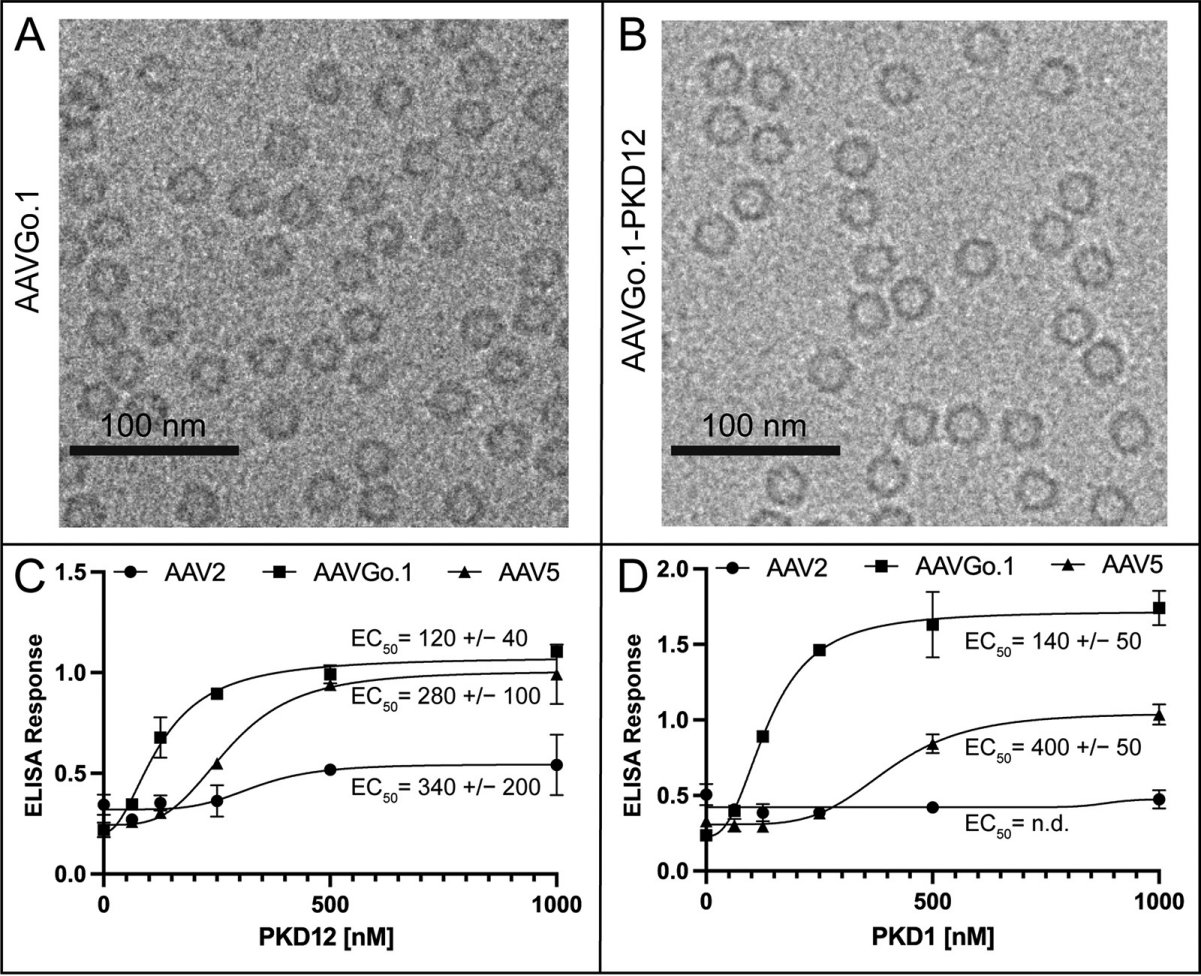
AAVGo.1-PKD12 complex binding avidity. (A) F30 TEM image of AAVGo.1 and (B) AAVGo.1-PKD12 complexes. (C and D) EC_50_ could be determined for AAV binding AAVR expression constructs with the exception of AAV2 with PKD1. GraphPad Prism 8 was used to fit curves by nonlinear regression and to calculate the EC_50_, shown with the 95% confidence limits.

AAVGo.1 virus-like particles (VLPs; without DNA genomes) were produced using Sf9 cells and examined via transmission electron microscope (TEM; Fig. 1A and B). AAVR binds to AAV5, AAVGo.1, and AAV2 with avidities that are similar order of magnitude, as measured by ELISA using nonglycosylated AAVR ectodomain constructs (Fig. 1C and D). In contrast to AAV2, the binding of both AAVGo.1 and AAV5 is mediated mostly through the PKD1 domain. Both have stronger binding avidity than AAV2, with AAVGo.1 nearly 2-fold stronger than AAV5 in PKD12 assays and approximately 3-fold stronger than AAV5 in PKD1 assays (Fig. 1C and D). PKD1 N395 is one of five N-linked asparagine glycosylation sites, and, although we know that glycosylation does not impact AAVR-binding (by viral overlay) and that N395A mutation does not appreciably affect AAV2 transduction (15), we cannot completely rule out subtle modulation of AAVR avidity mediated through glycosylation. Binding of AAVGo.1 to AAVR ectodomains via ELISA indicates that AAVGo.1 engages primarily with the PKD1 domain of AAVR, like AAV5, but with subtle differences warranting further investigation.

### The structure of AAVGo.1.

**FIG 2:**
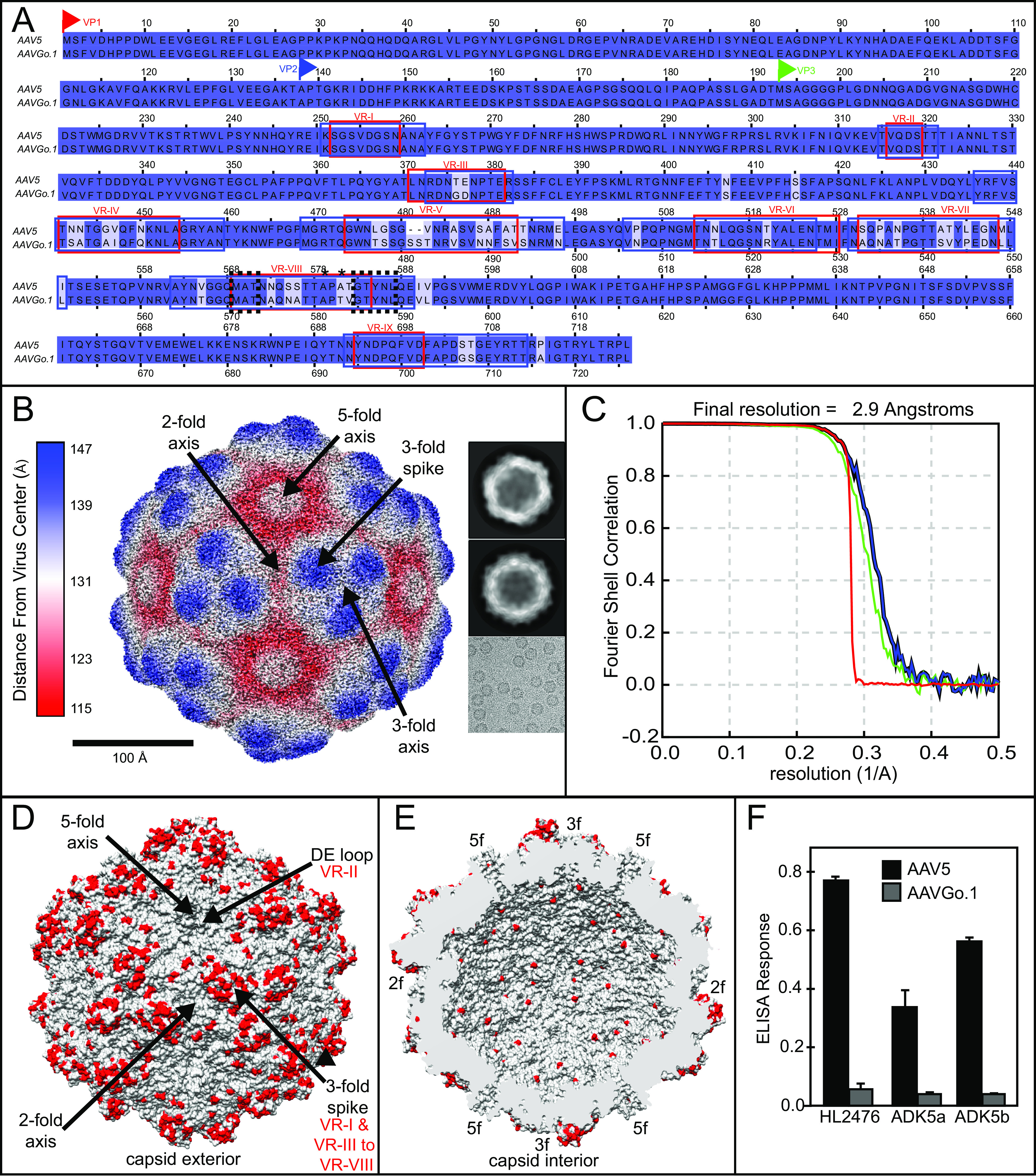
AAVGo.1 and AAV5 capsid amino acid variation affects antibody binding. (A) Amino acid alignment of AAVGo.1 and AAV5 VP1 amino acid sequences. Historic variable regions (VRs), as originally defined (25), are in red boxes, with more recently updated VR in blue boxes (26). Sialic acid binding site A (35), conserved in AAV5 and AAVGo.1, is outlined (black dotted lines) near VR-VIII. The A579T mutant (36) location and the A581T variant synonymous with a known point mutation (37) are labeled with asterisks. (B) Electron microscopy (EM) reconstruction of AAVGo.1 with two representative 2D classes and a sample cryo-EM micrograph image. (C) Fourier shell correlation (FSC) curves from reconstruction and postprocessing as described in Materials and Methods, green for unmasked maps, blue for masked, black for corrected maps, and red for phase-randomized masked maps. (D) The AAVGo.1 exterior surface representation with sequence differences (versus AAV5) (A) highlighted in red. (E) AAVGo.1 capsid interior surface with sequence differences (A) highlighted in red. (F) ELISA for AAV5 and AAVGo.1 using the anti-AAV5 monoclonal antibodies HL2476 (27), ADK5a, and ADK5b (38).

**FIG 3:**
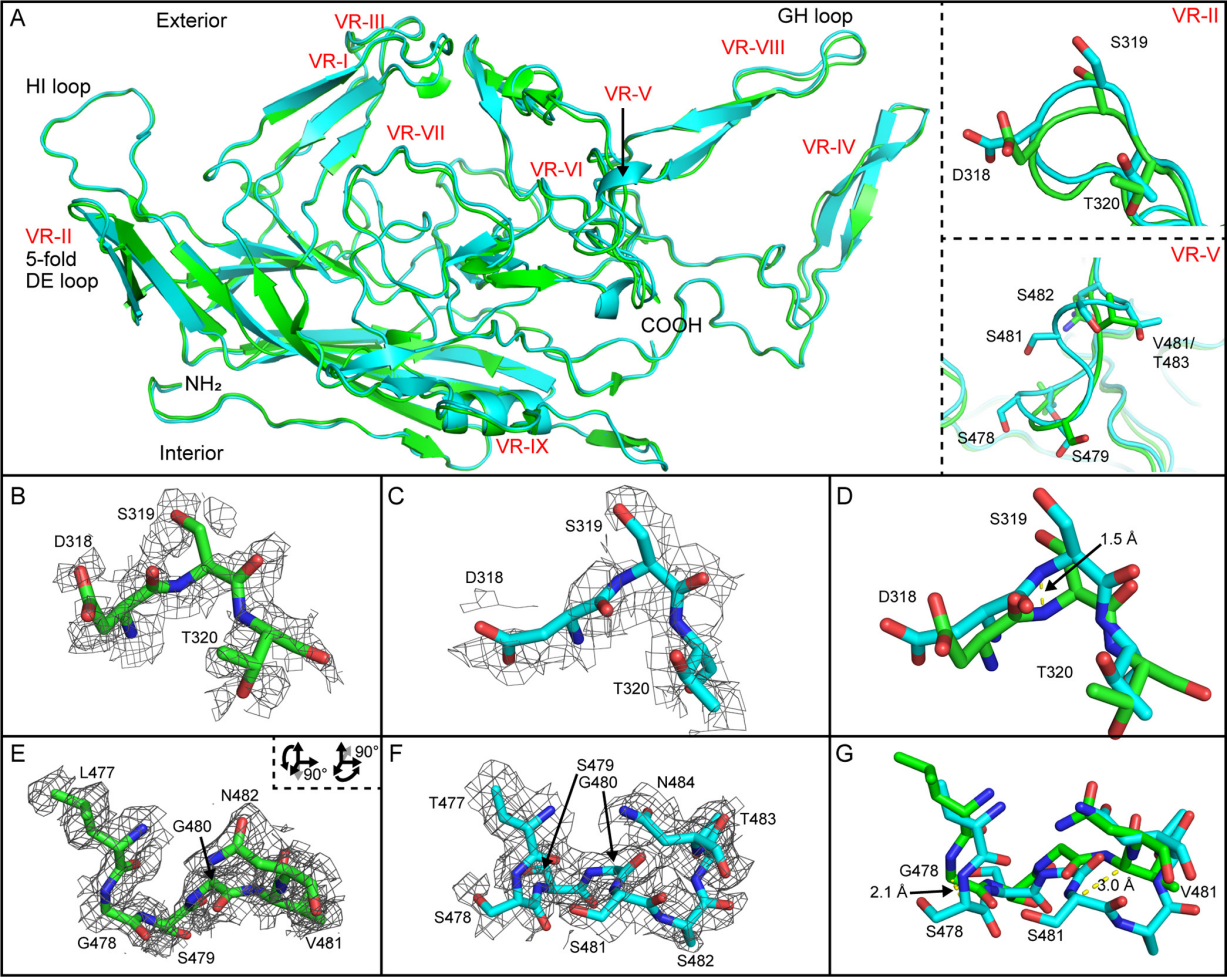
Comparison of the AAV5 and AAVGo.1 (uncomplexed) capsid structures. (A) Superposition of AAV5 (green) and AAVGo.1 (cyan) subunits. Upper right subpanel shows VR-II with select side chains. Lower right subpanel shows VR-V with its double serine insertion. (B) VR-II residues for AAV5 (green carbons) and (C) AAVGo.1 (cyan carbons) in their respective maps and then (D) from superimposed subunits. EM maps are contoured at 1.0 σ. The distance separating the overlaid atomic models is shown for panels D and G. (E) Residues from AAV5 VR-V in the map at 1.0 σ. (F) Corresponding AAVGo.1 residues in a map contoured at 1.5 σ. (G) VR-V residues structures superimposed.

Cryo-EM single particle analysis (SPA) yielded a reconstruction at 2.9 Å resolution for AAVGo.1 (Fig. 2). Sharpened maps provided detail for atomic modeling (Fig. 2) and were interpretable from residue 209 to 726 of the capsid protein in both reconstructions. The AAV capsid is composed of 3 viral proteins, VP1, VP2, and VP3, in icosahedral symmetry, in a relative ratio of 1:1:10 (21). By convention, the numbering of VP1 is used, even though it constitutes only 10% of the capsid subunits. The majority of the subunit common to VP1-3 is seen in the 60-fold averaged reconstructions, starting at residue 209 (Fig. 2 to 3). The N terminus of VP3 is residue 193 by VP1 numbering, so the atomic model accounts for 517 of 534 (96.8%) VP3 residues.

The AAVGo.1 capsid has a similar protein fold and surface topology to AAV5 structures (9, 11, 22). The capsid core, comprising a jellyroll fold found in many virus structures (23), and a single α-helix (αA), is the most conserved feature of AAVs, and AAVGo.1 is no exception. The AAVGo.1 core jellyroll has the standard topology with a backbone chain alternating between two stacked antiparallel β-sheets (almost rolling into a single tube). The sheet forming the inner surface of the capsid consists of β-strands B, I, D, and G that alternate with the “CHEF” strands of the outer sheet, together with a 5th inner strand, βA. βA is seen in many parvoviruses (24) at the edge of the BIDG sheet, running antiparallel to and turning directly into βB. Seven loops that connect strands of the BIDG and CHEF sheets encode much of the functionality of the capsid protein. Within the loops are regions of low-sequence conservation that decorate the outer surface of the virus, and these variable regions (VRs) are enumerated as VR-I to VR-IX (25, 26) (Fig. 2A and 3A). VR-I is found between βB and βC strands (BC loop), VR-II is found in the DE loop, VR-III is in the EF loop, VR-IV to VR-VIII are located in the GH loop, and the C terminus contains an additional VR-IX after the last βI strand. These VR loops serve as guideposts for understanding AAV-AAVR interactions (8 to 12).

AAVGo.1 and AAV5 capsid proteins have high homology (94%). All 42 amino acid differences occur in the VP3 region (more specifically the C terminal of AAVGo.1 VP1 residue 375), and the majority of these differences are located on the exterior of the capsid (19). Some of the 42 amino-acid differences occur within the footprint of known AAV5 antibodies, and ELISA antibody assays demonstrate that AAV5 antibodies bind AAV5 more than AAVGo.1 (Fig. 2F). The amino acid variability of AAV5 and AAVGo.1, therefore, has functional relevance to capsid-antibody interactions.

Minor structural differences are observed between AAV5 and AAVGo.1. The AAVGo.1 DE loop (VR-II), which forms the outer edge of the pore-like structure of the 5-fold axis, protrudes an additional 1.5 Å compared to AAV5 (Fig. 3A to D). A double serine insertion in VR-V of AAVGo.1 leads to two dislocations of 2.1 Å and 3.0 Å in the carbon backbone compared to AAV5 (Fig. 3E to G). The Coulombic potential of both AAV5 and AAVGo.1 bound to PKD1 (see below) show increased density in VR-II and VR-V compared to unbound capsids (not shown), thereby providing additional support for the structure. In conclusion, AAVGo.1 amino acid differences account for displacements up to 3.0 Å in VR-II and VR-V compared to the atomic structure of AAV5.

### Structure of AAVGo.1 and its AAVR receptor complex.

**FIG 4:**
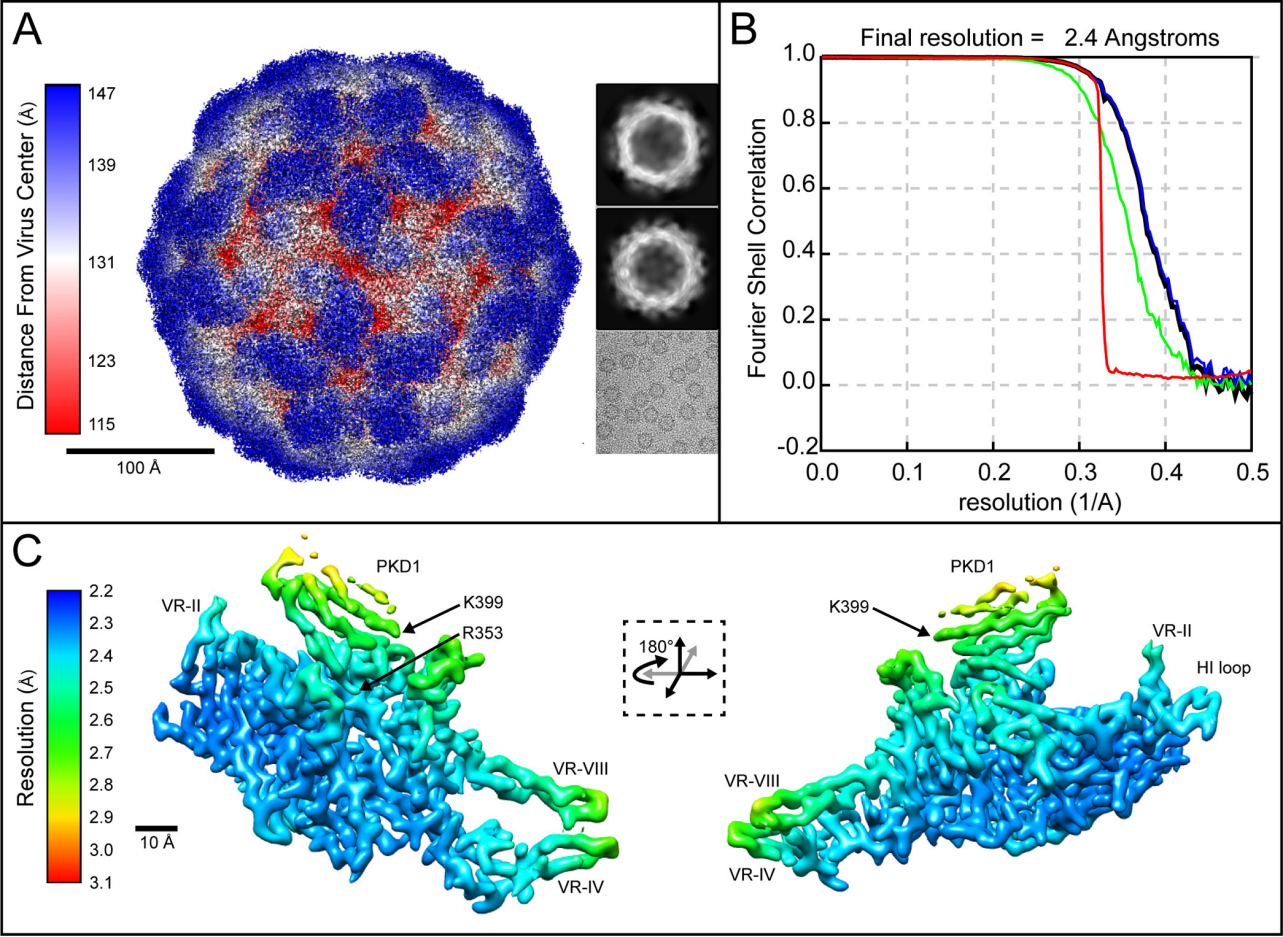
Cryo-EM reconstruction of AAVGo.1-PKD12 complexes. (A) AAVGo.1-PKD12 reconstruction with two representative 2D classes and a sample cryo-EM micrograph image. (B) Fourier shell correlation (FSC) curves from reconstruction and postprocessing as described in Materials and Methods, green for unmasked maps, blue for masked, black for corrected maps and red for phase randomized masked maps. (C) Local resolution of the AAVGo.1-PKD12 complex.

Cryo-EM single particle analysis (SPA) yielded reconstructions at 2.4 Å for the PKD12 complex (Fig. 4) that were interpretable within the capsid regions with AAVGo.1 (see above). The interactions between AAV5 and VR-IV, VR-VII, and VR-IX have been previously described (9, 11). The complex of AAVGo.1 bound to PKD12 differed in modest ways at the VR-VII and VR-IX interfaces. Just PKD1 was observed in the AAVGo.1 complex, with the two-domain PKD12 cryo-EM reconstruction and receptor density for the PKD1 subunit strongest at the viral interface (Fig. 4C). PKD1 density steadily weakens toward the 5-fold symmetr*y* axis of AAVGo.1, similar to previous cryo-EM studies of either PKD12 or PKD1-5 bound to AAV5 (9, 11).

**FIG 5:**
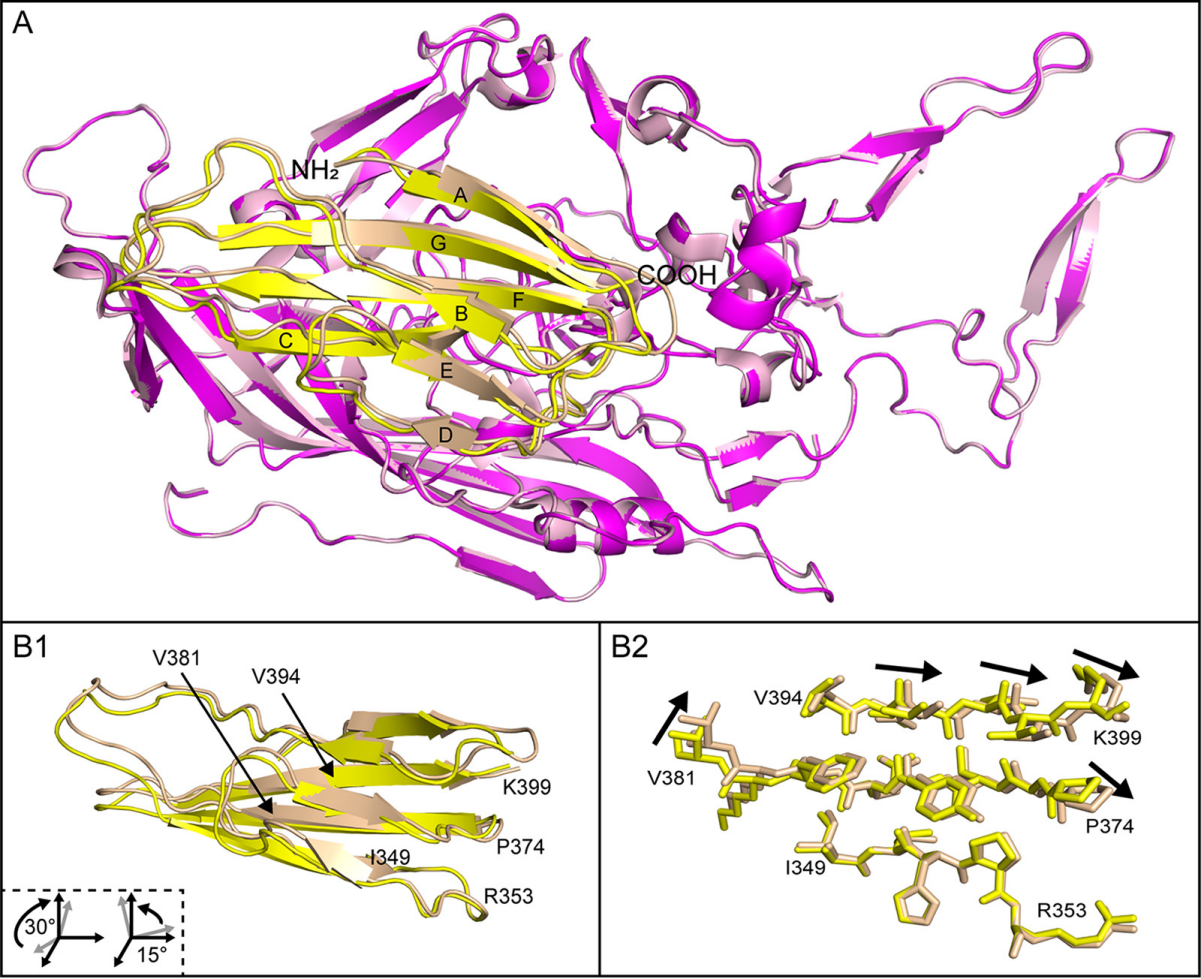
Comparison of the receptor-bound AAV5 and AAVGo.1 capsid structures. (A) Superposition of AAV5 (light pink) bound with PKD1 (wheat) and AAVGo.1 (magenta) bound with PKD1 (yellow). The β-strands of PKD1 are labeled alphabetically from A to G. (B) Alternate view of PKD1 (B1) with labeled amino acids corresponding to B2, which contains a magnified view of select amino acids. The arrows show directional vectors for the shift of PKD1 bound to AAV5 compared to PKD1 bound to AAVGo.1.

The steady weakening of map density away from PKD1 Arg_353_ seen in both AAV5 and AAVGo.1 complexes suggests the potential for (rigid) pivoting around the binding site. Receptor pivoting has not been observed in individual AAV5 or AAVGo.1 complexes, but when comparing PKD1 as bound to AAVGo.1 or AAV5, we see an overall rotation in the receptor position (Fig. 5). Displacements of ~1 Å are observed between PKD1 backbone carbons, and the shift appears to be a rotation around Arg_353_ that remains essentially unmoved (Fig. 5).

The PKD1 rotation results in an ~1 Å increase in the separation between the last amino acid resolved for PKD1 (Lys_399_) and the AAVGo.1 VR-IV loop. Contact has been observed between PKD1 Lys_399_ and VR-IV residues Asn_442_ and Asn_443_ of AAV5 (11). In AAVGo.1, however, these residues are replaced by Ser_442_ and Ala_443_, and interactions with PKD1 Lys_399_ are absent. Therefore, AAV5 VR-IV has unique contacts with the PKD1 C terminus absent in the AAVGo.1-PKD1 complex.

**FIG 6:**
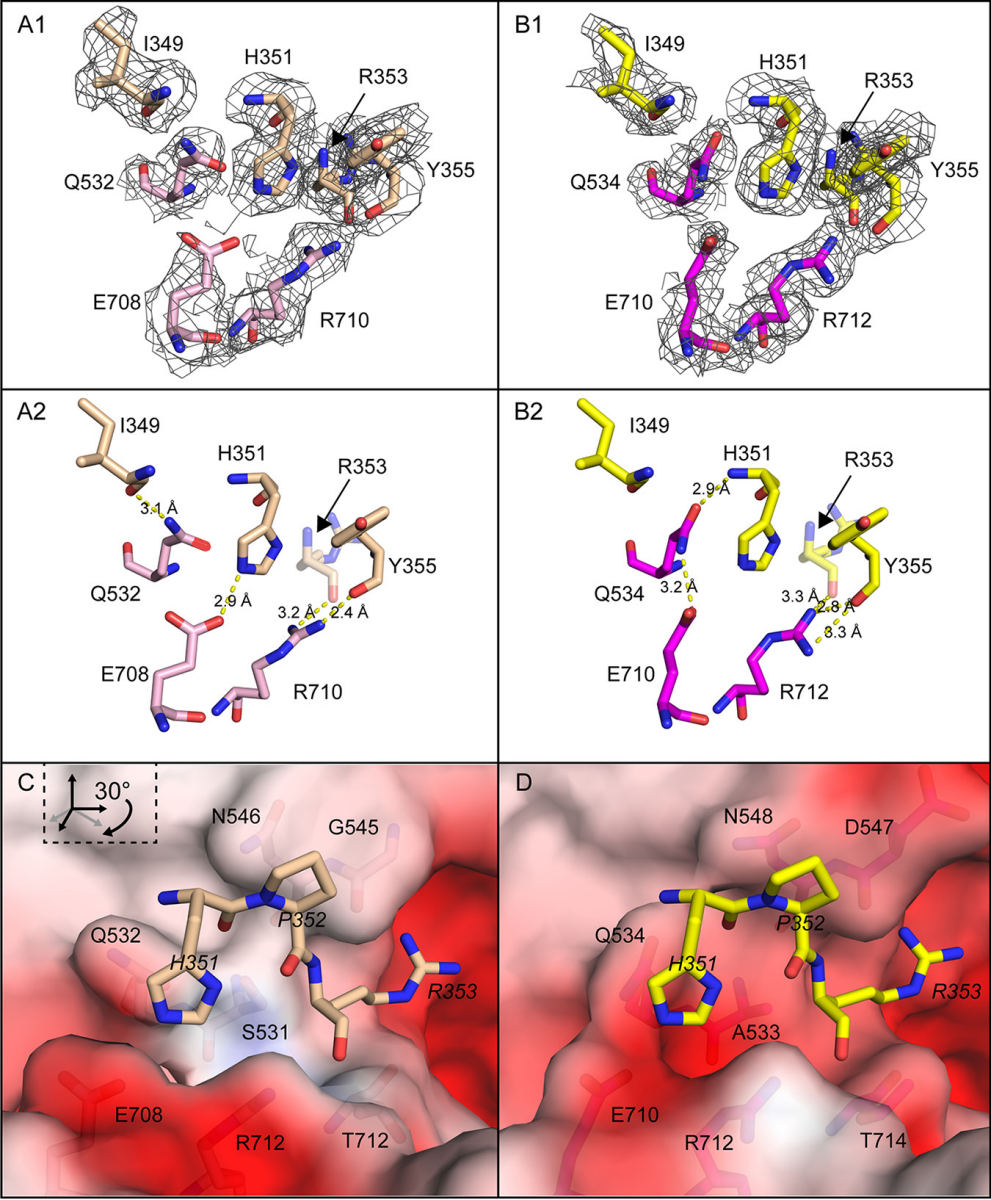
PKD1 H351 binding pocket interactions with AAV5 and AAVGo.1. (A) AAV5 H351 binding pocket with AAV5 carbons (light pink) and PKD1 (wheat). The Coulombic potential is contoured at 1.0 σ in panels A1 and B1. (B) Interactions near the AAVGo.1 H351 binding pocket, with AAVGo.1 carbons in magenta and PKD1 carbons in yellow. Panels C and D are rotated views of panels A and B, now with the solvent-excluded surfaces of AAV5 (C) and AAVGo.1 (D) colored by electrostatic potential from blue (most positive) to red (most negative). AAVR residues are in italics, virus residues in regular font. In both panels C and D, one can see complementarity with the basic residues of PKD1 (H351 and R353) nestled in negatively charged pockets. Electrostatic attraction is somewhat reduced with AAV5 (C) by the substitution of G545 for D547. Electrostatic potential was estimated from the uncomplexed virus structures using the Adaptive Poisson-Boltzmann Solver software (39).

**FIG 7:**
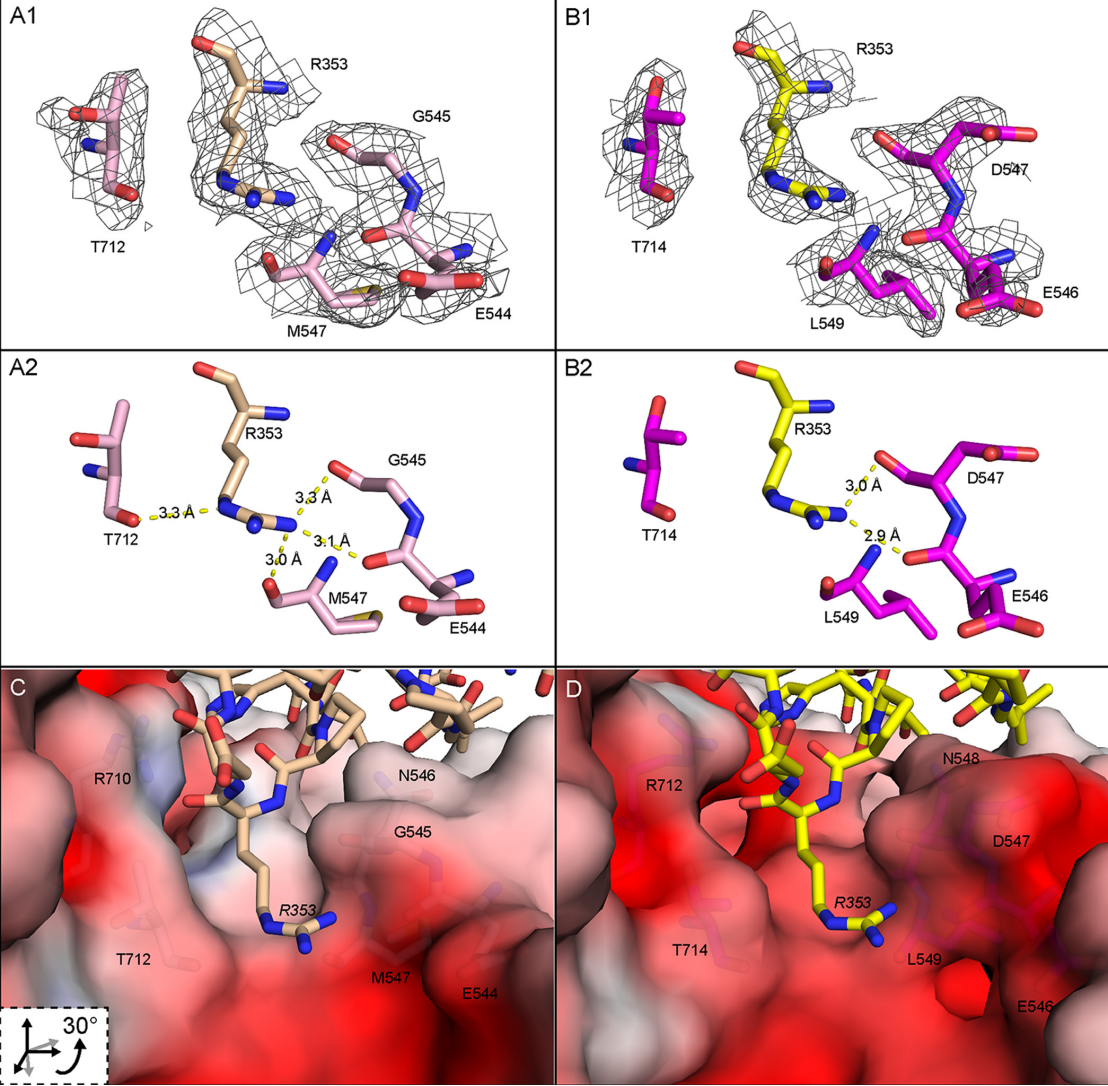
PKD1 R353 binding pocket interactions with AAV5 and AAVGo.1. (A) The AAV5 R353 binding pocket with AAV5 carbons (light pink) and PKD1 R353 (wheat). The EM maps for AAV are contoured at 1.5 σ in panels A1 and B1. (B) AAVGo.1 R353 binding pocket with AAVGo.1 carbons (magenta) and PKD1 R353 (yellow). Panels C and D are rotated views of panels A and B showing electrostatic and polar interactions of PKD1 at the AAV5 and AAVGo.1 binding subsites. (C) Detail of AAVR PKD1 (wheat carbons) binding to the AAV5 solvent-excluded surface, colored by electrostatic potential. (D) PKD1 (yellow carbons) binding to the AAVGo.1 electrostatic surface. Electrostatic potential was estimated using the Adaptive Poisson-Boltzmann Solver software (39).

Here, our attention is focused on the interaction of VR-VII and VR-IX residues with each other and with PKD1 within binding subsites. Central to the binding site are human PKD1 residues His_351_ and Arg_353_. The AAV5 VR-VII residue Gln_532_ makes contact with PKD1 (Ile_349_), and the Ile_349_ interaction is absent in AAVGo.1 (Fig. 6). The equivalent residue to AAV5 Gln_532_ is Gln_534_ in AAVGo.1, and AAVGo.1 Gln_534_ makes contact with both PKD1 His_351_ and AAVGo.1 VR-IX Glu_710_, leading to the formation of a less favorable binding pocket for PKD1 His_351_ in AAVGo.1. AAV5 Ser_531_, which is within 4 Å of the Arg_353_ backbone, increases the contact area compared to AAVGo.1 Ala_533_ (Fig. 6C). The AAV5 S531A substitution has also been shown to possibly increase AAV5 transduction relative to wild-type AAV5 in HEK293T cells (9). The substitution of AAV5 Gly_545_ (VR-VII) for AAVGo.1 Asp_547_ also affects PKD1 Arg_353_ binding subsite electrostatic interactions (Fig. 6 to 7). Furthermore, in AAV5 the Thr_712_ backbone carbonyl makes contact with PKD1 Arg_353_ Nε, which is absent in AAVGo.1 (Fig. 7). In summary, there are some detailed differences, the net effect of which is a modest change in contacts and polar interactions for the AAVGo.1 complex compared to AAV5 (Fig. 6 to 7).

**FIG 8:**
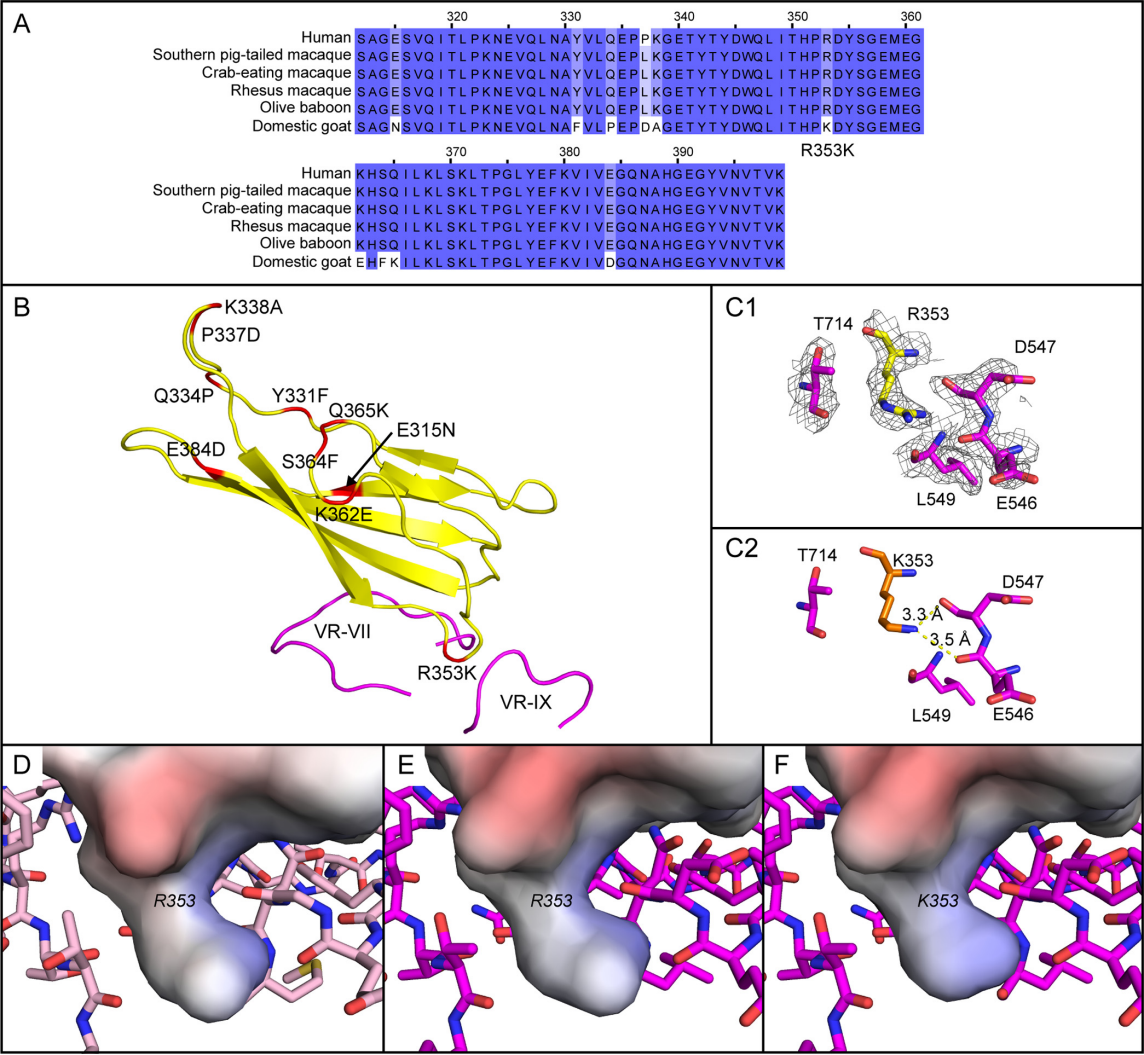
PKD1 amino acid variability. (A) Local sequence alignment of PKD1 from human, primate, and domesticated goat. (B) The structure of human PKD1 (yellow) as bound to AAVGo.1 with VR-VII and VR-IX capsid loops colored magenta. Variable PKD1 residues are shown in red. The binding interface is highly conserved, except for R353K. (C) The AAVGo.1 binding subsite for human PKD1 R353. (C1) Coulombic potential for the AAVGo.1/huAAVR complex is contoured at 1.5 σ. (C2) An atomic model for goat PKD1 can be built by homology with K353 overlaid on R353 of the human AAVR. (D) The electrostatic potential of AAV5-PKD1 is projected onto the solvent-excluded surface of the AAVR model and surrounded by interacting residues from AAV5 (pink carbons). (E) The surface of huAAVR is similarly colored by electrostatic potential, now calculated from the complex with AAVGo.1 (magenta carbons). (F) The surface of goat AAVR and its electrostatic interactions with AAVGo.1 are predicted by an R353K substitution in the model of AAVR. Electrostatic potentials were estimated using the Adaptive Poisson-Boltzmann Solver software (39).

An evaluation of PKD1 amino acid variability among humans, primates, and domesticated goats indicates goats have several variable sites (Fig. 8A). The binding interface is largely conserved, with the exception of an R353K substitution (Fig. 8B). The lysine can be modeled within the density for Arg_353_ in the map of the AAVGo.1-PKD12 receptor complex, suggesting that goat AAVR might be only minimally different and that Lys_353_ might have interactions similar to what we observe with human PKD1 Arg_353_ in the AAVGo.1 binding pocket (Fig. 7B and 8C). This suggests human AAV5 and domesticated goat AAVGo.1 receptor complex deviations near PKD1 Arg_353_ may reflect virus differences acquired via natural selection for host AAVR variation.

## DISCUSSION

The largest difference in sequence between AAVGo.1 and AAV5 is a tandem serine insertion in VR-V, consisting of a double insertion in AAVGo.1 relative to the AAV5 sequence L477(SS)G478. AAVGo.1 residues Thr477 to Ser482 are of below-average order in the sharpened maps of AAVGo.1 alone. In the AAVR complex, the AAVGo.1 map here is somewhat more ordered. Together, these indicate that the loop is intrinsically dynamic. The double serine insertion is within the epitope of AAV5-neutralizing monoclonal antibody MAb HL2476 (27), and if the Mab is representative of natural neutralizing polyclonal antibodies, it is plausible that the difference reflects selection through immune evasion. The loop that is extended by the insertion comes within 10 Å of PKD1, and we cannot rule out the possibility of an interaction with the unseen PKD1-2 interdomain linker, but there is no evidence of this from the structural studies.

Prior to this work, all complexes between AAV and AAVR were fundamentally similar to the AAV2 complex (8, 9, 12), with a single exception, AAV5 (9, 11). At first approximation, the binding of AAVGo.1 to AAVR is similar to that of AAV5, so the one exception becomes a second structural class in terms of the mode of binding. Between the two structural classes, the modes of binding are completely different. Within each class, the diversity in structure of the virus–receptor interface is much more modest. It is anticipated that within and between binding classes, variability at the sites of interaction could yield distinct transduction efficiencies, tissue tropism, and antibody neutralization profiles.

Within what we might now characterize as a PKD1-binding clade of AAVs, structural differences in the AAVR complexes of AAV5 and AAVGo.1 are minimal. There is an overall rotation of the PKD1 domain, but otherwise differences are very subtle, consistent with modest differences in transduction efficiencies. It is plausible that the structural differences observed could account for different transduction efficiencies, but explicit associations would be somewhat speculative at this time. Many factors beyond avidity affect transduction. Furthermore, avidity is the result not only of the interactions seen, but from free energy changes in protein order or solvation upon receptor binding that are not quantifiable by cryo-EM.

At a more qualitative level, it is an intriguing possibility that the avidity of the complex between human AAV5 and AAVR is not fully optimized, as suggested by tighter binding of a domesticated goat AAV. Is this due to compromise between receptor binding and immune neutralization escape? Or is evolutionary selection of high avidity limited by another uncharacterized step in the virus’s life cycle where dissociation from AAVR is needed?

## MATERIALS AND METHODS

### Expression and purification of AAVGo.1 and AAVR constructs.

AAV2 (0.9 mg/mL) and AAV5 (7.6 mg/mL) virus-like particles (VLPs) were produced using Sf9 cells and purified as previously described (10, 11). AAVGo.1 was cloned as previously described (28), produced using Sf9 cells, and purified via ultracentrifugation in a cesium chloride gradient. Gradient ultracentrifugation was repeated 4 times, yielding a final concentration of 38.8 mg/mL. Sample purity and concentration were evaluated using nanodrop, SDS-PAGE, and negatively stained electron microscopy with a JEOL JEM-1400 120 kV transmission electron microscope (TEM). AAVR fragment constructs (His_6_PKD1, His_6_PKD12, and His_6_PKD15) were expressed and purified as previously described (10).

### Enzyme-linked immunosorbent assay (ELISA).

For the PKD1 and PKD12 binding assays, ELISAs were carried out in duplicate using a modified direct ELISA protocol (6, 11). AAV2, AAV5, or AAVGo.1 VLPs (2.5 μg/mL) were incubated in ELISA plates (Corning Costar number 9018) in 100 mM NaHC03 (pH 9.6) and washed with TBST buffer (0.05% TWEEN in TBS [Tris-buffered saline]). Plates were incubated with N-terminally His-tagged AAVR (His_6_PKD1, His_6_PKD12, or His_6_PKD15) and detected with anti-6× His tag horseradish peroxidase (HRP) (Abcam number ab1187) antibody. 3,3′5,5′-Tetramethylbenzidine (TMB) ELISA Substrate (Abcam number ab171523) was added to each well, and development was stopped using 1 M hydrochloric acid. The optical density of plates was evaluated at 450 nm using a microplate reader (BioTek Synergy H1 Hybrid Multi-Mode Reader). Curves were fitted via nonlinear regression, and the 50% effective concentrations (EC_50_) were calculated using GraphPad Prism 8, using the 95% confidence limits as error estimates.

For the antibody binding assays, AAV5 and AAVGo.1 (50 μL at 3 μL/mL in 100 mM sodium bicarbonate buffer at pH 9.6) were incubated in multiple wells of a 96-well plate (Costar, Corning USA, Corning, NY, USA, cat. no. 9018) overnight on a rocking platform. The wells were then washed three times with Tween 20 Tris-buffered saline (TTBS; 0.05% vol/vol Tween 20, 50 mM Tris-HVL, 150 mM sodium chloride at pH 7.5) followed by blocking with bovine serum albumin (BSA; Fisher Scientific, Hampton, NH, USA, cat. no. BP9703-100; 150 μL at 3% wt/vol in TTBS) and incubated for 1 h. Unbound BSA was removed via three additional washes with TTBS. One hundred microliters of antibodies, diluted in TTBS, were added and incubated for 1 h at the following final concentrations: 2 μg/mL of HLA2476 (Sigma-Aldrich, Temecula, USA, cat. no. MABF2768), 100 μg/mL of ADK5a (Progen, Heidelberg, Germany, cat. no. 615148), and 4 μg/mL of ADK5b (Origene, Rockville, MD, USA, cat. no. AM09121PU-N). After 1 h, unbound antibodies were removed and the wells were washed three times with TTBS. One hundred microliters of a secondary HRP-conjugated, goat-derived, anti-mouse antibody (Alexa Fluor, Thermo Fisher Scientific, Waltham, MA, USA, cat. no. A28175) were then added at 4 μg/mL and incubated for 1 h. Unbound secondary antibody was removed via washing three times with TTBS. Ninety microliters of TMB ELISA substrate were then added for 5 to 10 min. The reaction was quenched with 80 μL of 1 M HCL, and the absorbance was measured at 450 nm using a plate reader.

### TEM/single particle cryo-EM data acquisition and 3D image reconstruction.

AAVGo.1 and AAVGo.1-PKD12 complexes were prepared and imaged as previously described for AAV2 and AAV5 (11, 12). Briefly, AAVGo.1 was dialyzed/diluted into HN buffer (25 mM HEPES pH 7.4 and 150 mM NaCl) at 0.33 mg/mL and PKD12 at 0.75 mg/mL. Grids had an ultrathin continuous carbon film layered on Lacey carbon supports (400 mesh; copper; Ted Pella number 01824). The grids were glow discharged using a PELCO easiGlow Glow Discharge Cleaning System. After application of 2 μL of sample, AAVGo.1 grids were blotted once and plunged directly into liquid ethane using an FEI Mark IV Vitrobot (force = 4, time = 2, temperature = 25, humidity = 100). PKD12 complex grids were prepared by adding 2 μL AAVGo.1 to the grid with a 2-min incubation time. The grid was then wicked with Whatman filter paper (grade 595), and 2 μL of receptor was immediately added followed by blotting and plunging via Vitroblot (force = 4, time = 3, temperature = 25, humidity = 100).

Cryo-EM grids were screened using an FEI Tecnai F30 Twin 300 kV TEM in preparation for single-particle Cryo-EM. AAVGo.1, and AAVGo.1-PKD12 complexes were imaged using an FEI Titan Krios equipped with a Gatan K3 digital camera in superresolution mode at a dose rate of 40 frames per 3.12 s exposure(Table 1). Automated micrograph data collection was enabled using Leginon (29).

**TABLE 1:** Cryo-EM data acquisition and processing

	AAVGo.1	AAVGo.1-PKD12
Data collection		
Magnification (×)	64,000	64,000
Voltage (kV)	300	300
Electron exposure (e/Å^2^) per micrograph	35.4	32.9
Frames/micrograph	40	40
Defocus range (μm)	−0.7 to −2.0	−0.7 to −2.0
Pixel size (Å)	0.664	0.664
Data processing		
Motion correction	Relion 3.1.1	Relion 3.1.1
CTF estimation	CTFFIND-4.1	CTFFIND-4.1
Symmetry imposed	I1	I1
Initial particle images	246,094	208,921
Final particle images	140,568	71,095
Map resolution	2.93	2.36
FSC threshold	0.143	0.143

Relion 3.1.0 was used to process AAVGo.1 (30). Seven hundred thirteen micrographs were motion corrected using MOTIONCOR2 (31), and CTFFind-4.1 was used for CTF correction (32). A total of 246,094 particles were picked with Relion’s Autopick, which were culled down to 188,650 through several rounds of 2D classification. An additional round of 3D classification (symmetry group C1) yielded 140,568 particles. Per-particle CTF and Bayesian polishing followed by another round of 3D refinement (I1) produced an unmasked map of 2.93 Å, and masking generated a final map of 2.91 Å (gold-standard FSC_0.143_) (Fig. 2).

Relion 3.1.1 was used for the complex of PKD12 with AAVGo.1 (30). A total of 1,204 micrographs were motion corrected with MOTIONCOR2 (31), and CTF estimation was carried out using CTFFind-4.1 (32). From 1,127 micrographs, 208,921 particles were picked using Relion’s Autopick with the AAVGo.1 structure as a template. We carried out 3D refinement directly on the extracted particles, and 3D classification was used with C1 symmetry imposed, resulting in 71,095 particles for 3D auto-refinement. Per-particle CTF and motion correction were performed followed by an additional round of 3D auto-refinement with I1 symmetry, resulting in an unmasked structure at 2.55 Å. Masking resulted in a final resolution of 2.36 Å (gold-standard FSC_0.143_; Fig. 4).

### Model building and structure refinement.

A preliminary model for AAVGo.1 was generated using the AAVGo.1 VP3 protein sequence threaded into the 2.1 Å AAV5 structure (PDB ID: 7kp3) in Coot (33). The starting model for the PKD1 domain of the receptor came from the 2.5 Å AAV5-PKD12 structure (PDB ID: 7kpn). The models and imaging parameters were refined using RSRef 0.5.6 (34) with a final real-space correlation coefficient (CC) of 0.87 for AAVGo.1 and 0.83 for complexed AAVGo.1-PKD12. (Correlation coefficients were calculated using all map grid points within 2.0 and 2.4 Å of all atoms, respectively.) Manual model adjustments were executed in Coot 0.8.9.2.

### Data availability.

Atomic models and cryo-EM maps are available at the PDB (ID 7TI4 for AAVGo.1 and ID 7TI5 for the complex) and EMDB (ID EMD-25909 for AAVGo.1 and ID EMD-25910 for the complex).
